# COPD-Type lung inflammation promotes K-ras mutant lung cancer through epithelial HIF-1α mediated tumor angiogenesis and proliferation

**DOI:** 10.18632/oncotarget.26030

**Published:** 2018-08-31

**Authors:** Maria Miguelina De la Garza, Amber M. Cumpian, Soudabeh Daliri, Susana Castro-Pando, Misha Umer, Lei Gong, Nasim Khosravi, Mauricio S. Caetano, Marco Ramos-Castañeda, Alejandra Garza Flores, Evelyn C. Beltran, Hai T. Tran, Michael J. Tuvim, Edwin J. Ostrin, Burton F. Dickey, Christopher M. Evans, Seyed Javad Moghaddam

**Affiliations:** ^1^ Department of Pulmonary Medicine, The University of Texas M. D. Anderson Cancer Center, Houston, Texas, USA; ^2^ Tecnológico de Monterrey, Escuela de Medicina y Ciencias de la Salud, Monterrey, Nuevo León, Mexico; ^3^ Tianjin Lung Cancer Institute, Tianjin Medical University, Tianjin, China; ^4^ Department of Thoracic Head and Neck Medical Oncology, The University of Texas M. D. Anderson Cancer Center, Houston, Texas, USA; ^5^ Department of General Internal Medicine, The University of Texas M. D. Anderson Cancer Center, Houston, Texas, USA; ^6^ Division of Pulmonary Sciences and Critical Care Medicine, University of Colorado Denver School of Medicine, Aurora, Colorado, USA; ^7^ The University of Texas M.D. Anderson Cancer Center UTHealth Graduate School of Biomedical Sciences, Houston, Texas, USA

**Keywords:** lung cancer, COPD, HIF-1, inflammation, K-ras

## Abstract

Chronic obstructive pulmonary disease (COPD), an inflammatory disease of the lung, is an independent risk factor for lung cancer. Lung tissues obtained from human smokers with COPD and lung cancer demonstrate hypoxia and up-regulated hypoxia inducible factor-1 (HIF-1). HIF-1 activation is the central mechanism for controlling the cellular response to hypoxia during inflammation and tumor development. These facts suggest a link between COPD-related airway inflammation, HIF-1, and lung cancer. We have previously established a mouse model of COPD-like airway inflammation that promotes lung cancer in a K-ras mutant mouse model (CC-LR). Here we show that tumors in the CC-LR model have significantly elevated levels of HIF-1α and HIF-1 activity. To determine the tumor-promoting functions of HIF-1 in CC-LR mice, the gene *Hif1a* which encodes HIF-1α and is required for HIF-1 activity, was disrupted in the lung epithelium of CC-LR animals. Airway epithelial specific HIF-1α deficient mice demonstrated significant reductions in lung surface tumor numbers, tumor angiogenesis, and tumor cell proliferation in the absence or presence of COPD-like airway inflammation. In addition, when CC-LR mice were bred with transgenic animals that overexpress a constitutively active mutant form of human HIF-1α in the airway epithelium, both COPD- and adenocarcinoma-like phenotypes were observed. HIF-1α overexpressing CC-LR mice had significant emphysema, and they also showed potentiated tumorigenesis, angiogenesis, and cell proliferation accompanied by an invasive metastatic phenotype. Our gain and loss of function studies support a key role for HIF-1α in the promotion of lung cancer by COPD-like inflammation.

## INTRODUCTION

Cigarette smoking is the principal cause of chronic obstructive pulmonary disease (COPD) and lung cancer [1, 2]. Although lung cancer is primarily caused by the induction of DNA mutations in lung epithelial cells, several studies have suggested that COPD-related risk for lung cancer is related to COPD-driven inflammation independently of cigarette smoke-induced mutagenesis [3–7]. Repeated cycles of respiratory tract epithelial mucosal damage and repair in response to chronic cigarette smoke exposure and/or infection in COPD can result in epithelial hyperplasia and metaplasia [8]. Although metaplasia is initially an adaptive response to persistent irritation, clinical surveillance has demonstrated that lung cancer often develops within metaplastic microenvironments [9]. Under non-neoplastic conditions, hyperplastic lesions can be induced transiently by inflammation or injury. However, when these lesions transit from having poorly controlled to uncontrolled proliferation, they take on neoplastic and atypical adenomatous appearances. The neoplastic transformation may be further accompanied by the acquisition of the growth, angiogenic, invasive, and metastatic characteristics of malignancy [10].

Due to the expansive activities within neoplastic structures, tumor cells are highly metabolic. However, they are poorly vascularized as they initially form, resulting in increased O_2_ demand amidst inadequate O_2_ supply. Accordingly, tumor cells experience significant hypoxia and they adapt to this environment by two means: up-regulation of anaerobic metabolic pathways (demand control) and up-regulation of pro-angiogenic pathways (supply control) [11–13]. Similarly, sites of inflammation (benign or neoplastic) are also characterized by local hypoxia and high glycolytic demands [14, 15]. Under neoplastic and non-neoplastic conditions, hypoxic cells attempt to restore oxygen supply and demand homeostasis by activating compensatory response programs. A central mechanism for the cellular response to hypoxia occurs through regulation and activation of hypoxia inducible factor-1 (HIF-1) [16]. HIF-1 is a transcription factor that plays central roles in crucial aspects of carcinogenesis, including angiogenesis, cell survival, glucose metabolism, invasion, and metastasis. HIF-1 is a basic helix-loop-helix transcription factor [17] comprised of α and β subunits. HIF-1β is constitutively produced but is inactive in the absence of HIF-1α, whose levels are directly controlled by oxygen levels, with normally high levels of oxygen causing proteolytic degradation of HIF-1α. Cellular hypoxia results in stabilization of HIF-1α, the formation of stabilized HIF-1α/HIF-1β heterodimers, and the expression of HIF-target genes. In addition to increases in HIF-1 observed in lung cancer cells [18–20], hypoxia and up-regulated HIF-1α have been shown in airway tissues obtained from human smokers with COPD [19], suggesting a potential role for inflammation-mediated hypoxia and HIF-1 pathway activation in the progression from COPD to lung cancer.

We have previously established a mouse model of COPD-like airway inflammation induced by repetitive exposure to an aerosolized lysate of non-typeable (i.e., unencapsulated) *Hemophilus influenzae* (NTHi), which commonly and persistently colonizes airways of COPD patients [21]. We have shown that this type of airway inflammation promotes lung cancer in a K-ras mutant mouse model [22]. NTHi-driven tumor promotion is associated with significant up-regulation of HIF-1α, and HIF-1 target genes [23], suggesting that HIF-1 activation during COPD-like inflammation that is independent of tobacco smoke may provide a crucial link between COPD and lung cancer.

To test whether COPD-like lung inflammation, and HIF-1 activation are linked causatively to lung cancer promotion, we studied the role of the HIF-1α pathway using genetic targeting of this pathway in airway epithelial cells in mice *in vivo*. We found that COPD-like inflammation and HIF-1α overexpression are each sufficient to potentiate K-ras induced lung tumorigenesis, and that HIF-1α expression in the airway epithelium is required for tumor promotion, increased tumor angiogenesis, and cell proliferation.

## RESULTS

### Lung cancer progression is associated with increased angiogenesis and activation of the HIF-1α pathway

**Figure 1:**
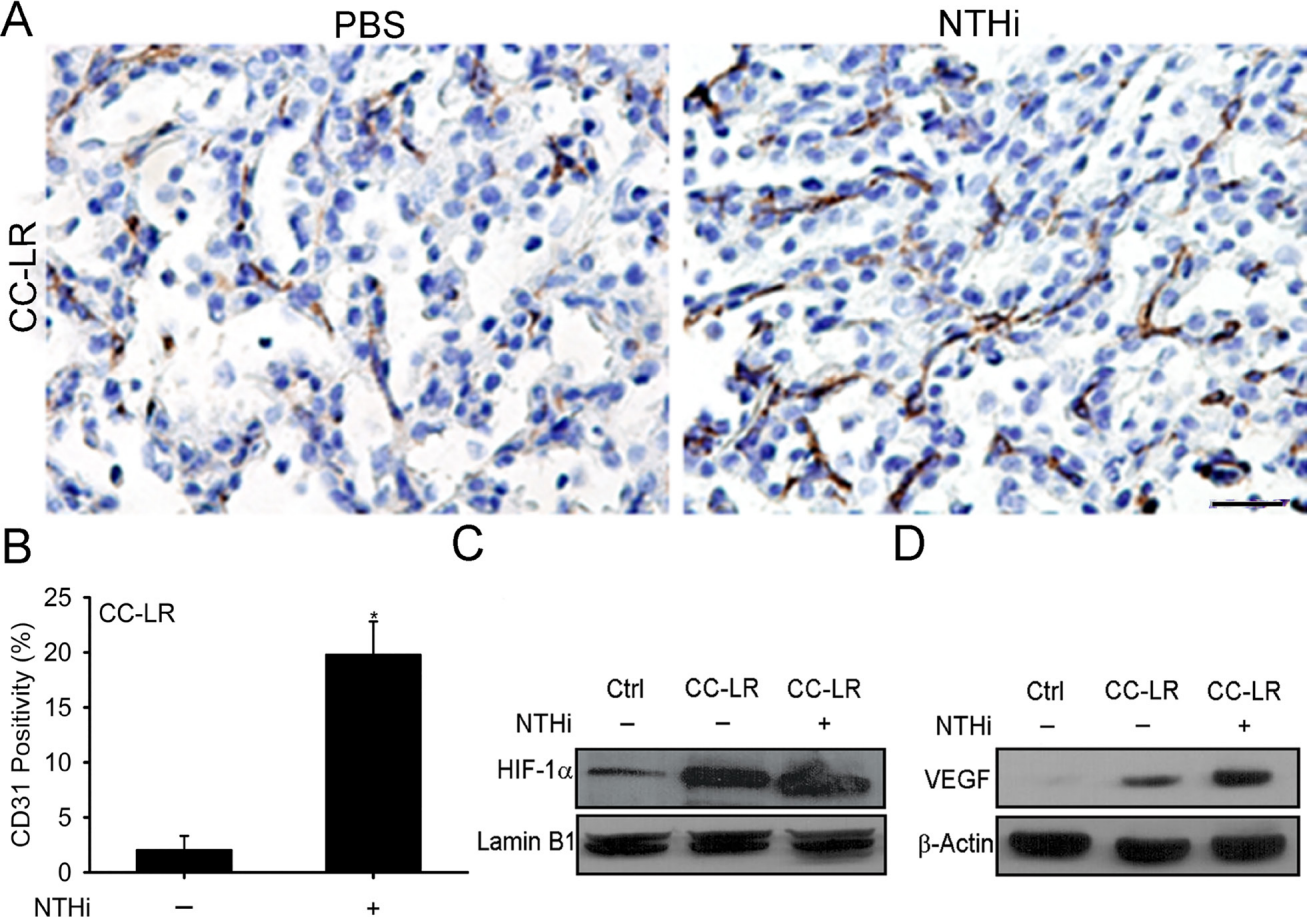
Promotion of K-ras induced lung tumorigenesis by inflammation is associated with increased angiogenesis and activation of the HIF-1α pathway (**A**) Representative photomicrographs of CD31 immunolabeled lung tumor tissues of 14 weeks old CC-LR mice in the absence or presence of NTHi-induced COPD-type airway inflammation (40× magnification, scale bar = 100 μm, applicable to all panels). (**B**) Quantitative analysis of CD31 positive staining in lung tissue of CC-LR mice in the absence or presence of NTHi exposure (mean ± SE; ^*^ = *P* ≤ 0.05 for CC-LR vs CC-LR plus NTHi; *n* = 3). (**C**) Western blot analysis of HIF-1α in the whole lung tissue nuclear extract. (**D**) Western blot analysis of VEGF in protein extracted from whole lung tissue.

We have previously found that NTHi-induced COPD-like airway inflammation upregulates HIF-1α and vascular endothelial growth factor (VEGF) gene expression in the whole lung of a K-ras mutant mouse model of lung cancer (CC-LR or CCSP^Cre^/LSL–Kras^G12D^ mouse) [23]. These two genes (*Hif1a, and Vegf)* have an important role in the regulation of angiogenesis, and we found significant angiogenesis in lung tumor tissues from CC-LR mice with NTHi-induced COPD-like inflammation (Figure 1A, 1B, and Supplementary Figure 1). This was associated with increased expression levels of HIF-1α in nuclear extract of whole lung tissue lysates (Figure 1C), and VEGF in whole lung tissue lysates (Figure 1D) from CC-LR mice. These data further suggest a strong association between COPD-like lung inflammation, HIF-1α activity, angiogenesis and lung cancer promotion.

### Lack of HIF-1α in the airway epithelium suppresses lung cancer promotion

**Figure 2:**
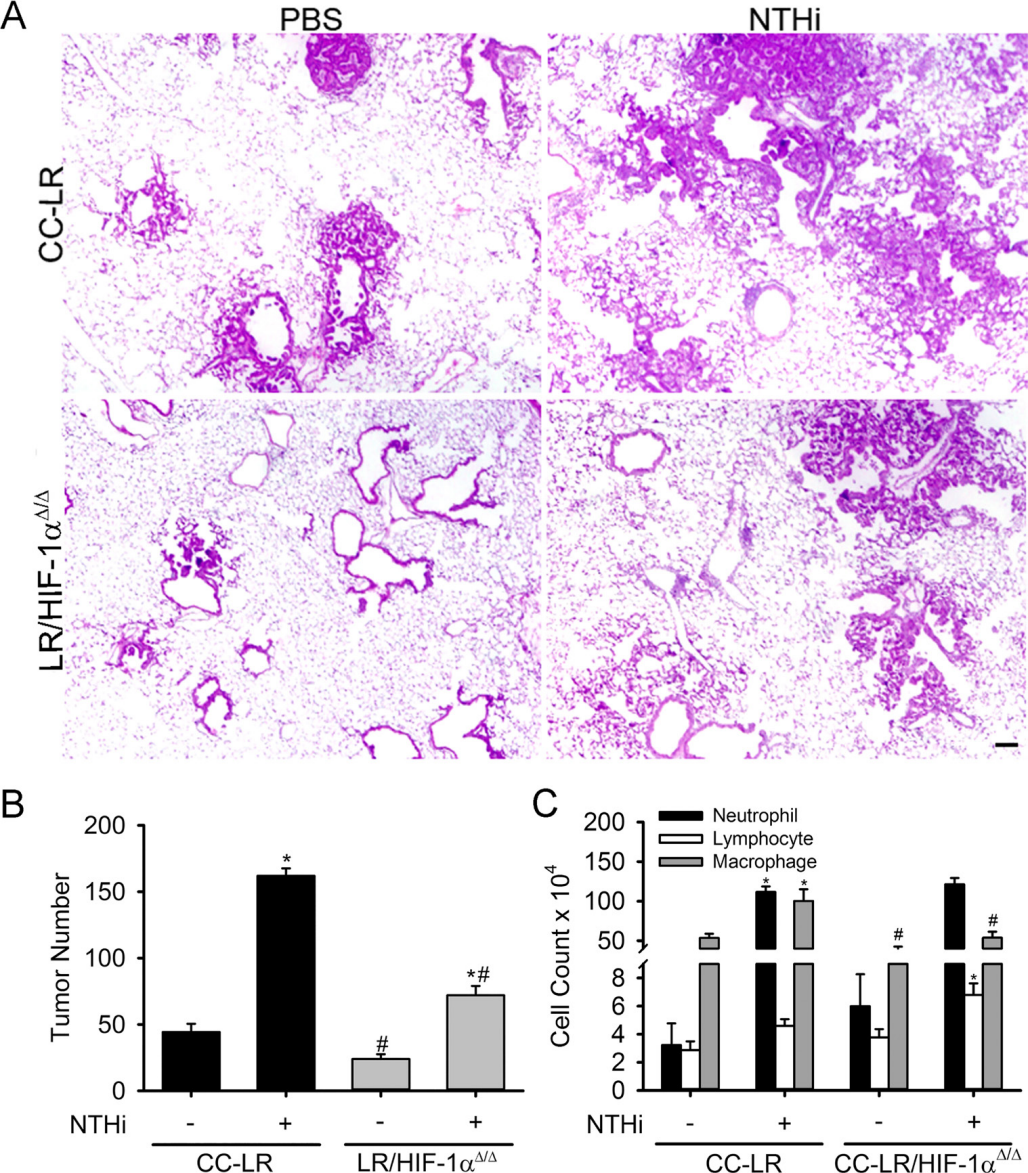
Lack of HIF-1α in the airway epithelium suppresses K-ras induced lung tumorigenesis and its promotion by inflammation CC-LR and LR/HIF-1α^Δ/Δ^ mice were exposed to an NTHi lysate aerosol starting at age 6 weeks weekly for 8 weeks to induce COPD-like airway inflammation. (**A**) Histopathological appearance of lung tissue from CC-LR and LR/HIF-1α^Δ/Δ^ in the absence or presence of COPD-type airway inflammation at age of 14 weeks (4× magnification, scale bar = 10 μm, applicable to all panels). (**B**) Lung surface tumor number in CC-LR and LR/HIF-1α^Δ/Δ^ mice in the absence or presence of COPD-type airway inflammation at age of 14 weeks (mean ± SE; ^*^, *P* < 0.05 for CC-LR or LR/HIF-1α^Δ/Δ^ with NTHi exposure vs. without NTHi exposure; ^#^, *P* < 0.05 for CC-LR without NTHi exposure vs. LR/HIF-1α^Δ/Δ^ without NTHi exposure or CC-LR with NTHi exposure vs. LR/HIF-1α^Δ/Δ^ with NTHi exposure; *n* = 12). (**C**) Lineage-specific leukocyte numbers in BALFs of CC-LR and LR/HIF-1α^Δ/Δ^ mice 1 day after last NTHi aerosol exposure at age of 14 weeks (mean ± SE; ^*^, *P* < 0.05 for CC-LR or LR/HIF-1α^Δ/Δ^ with NTHi exposure vs. without NTHi exposure; ^#^, *P* < 0.05 for CC-LR without NTHi exposure vs. LR/HIF-1α^Δ/Δ^ without NTHi exposure or CC-LR with NTHi exposure vs. LR/HIF-1α^Δ/Δ^ with NTHi exposure; *n* = 6).

In order to study the causal role of HIF-1α in lung cancer promotion, we have targeted its expression in the airway epithelium of the CC-LR mouse by crossing this mouse to a conditional knock out mouse with both alleles of exon 2 of *Hif1a* flanked by loxP sites [24]. This resulted in a mouse that specifically expresses a mutant Kras allele while lacking HIF-1α activity in the airway epithelium (LR/HIF-1α^Δ/Δ^) because of the club cell secretory protein (CCSP) promoter which is directing Cre expression to airway secretory cells (club cells). This resulted in a ~50% (1.8-fold) reduction in lung surface tumor number compared to age and sex-matched control CC-LR mice (44 ± 6 in CC-LR vs 24 ± 3 in LR/HIF-1α^Δ/Δ^) (Figure 2A, 2B). HIF-1α deficiency in the airway epithelium also significantly reduced the number of visible tumors on the lung surface of CC-LR mice by >50% (2.2-fold) after inducing COPD-like airway inflammation using 8 weekly NTHi lysate exposures (162 ± 5 in CC-LR NTHi treated vs 72 ± 7 in LR/HIF-1α^Δ/Δ^ NTHi treated) (Figure 2A, 2B). Histopathologic examination of the lung from LR/HIF-1α^Δ/Δ^ mice also showed less tissue inflammation and lower numbers of advanced adenomatous lesions compared to CC-LR mice (Figure 2A). Noticeably, HIF-1α deficiency in the airway epithelium changed the bronchoalveolar lavage fluid (BALF) cellular component of CC-LR mice mostly by reducing the recruitment of macrophages into the lung at baseline and in response to NTHi exposure (Figure 2C). There were no significant changes in neutrophil or lymphocyte populations. Reduced macrophage number was associated with a significant reduction in levels of the pro-inflammatory cytokine IL-6 and chemokine KC (Table 1).

**Table 1:** Selected cytokine and chemokine levels in bronchoalveolar lavage fluid

Cytokine	IL-6		KC	
Mouse Group	− NTHi	+ NTHi^*^	− NTHi	+ NTHi^*^
**CC-LR**	19.9 ± 1.6	131 ± 3.0	29.7 ± 8.9	126.7 ± 9.4
**CC-LR/HIF-1α^Δ/Δ^**	2.09 ± 0.6	44.55 ± 12.9	16.98 ± 7.5	51.06 ± 10.8
**CC-LR/HIF-1α Tg**	61.61 ± 9.2	N/A	33.7 ± 5.6	N/A

Abbreviations: NTHi, nontypeable *Haemophilus influenza*; IL-6, interleukin 6; KC, keratinocyte-derived chemokine, N/A, not applicable.

All data are the mean ± SEM and are expressed as pg/ml (*n* = 4).

^*^Bronchoalveolar lavage fluids were collected at the age of 14 weeks and the first day after 8th NTHi exposure.

**Figure 3:**
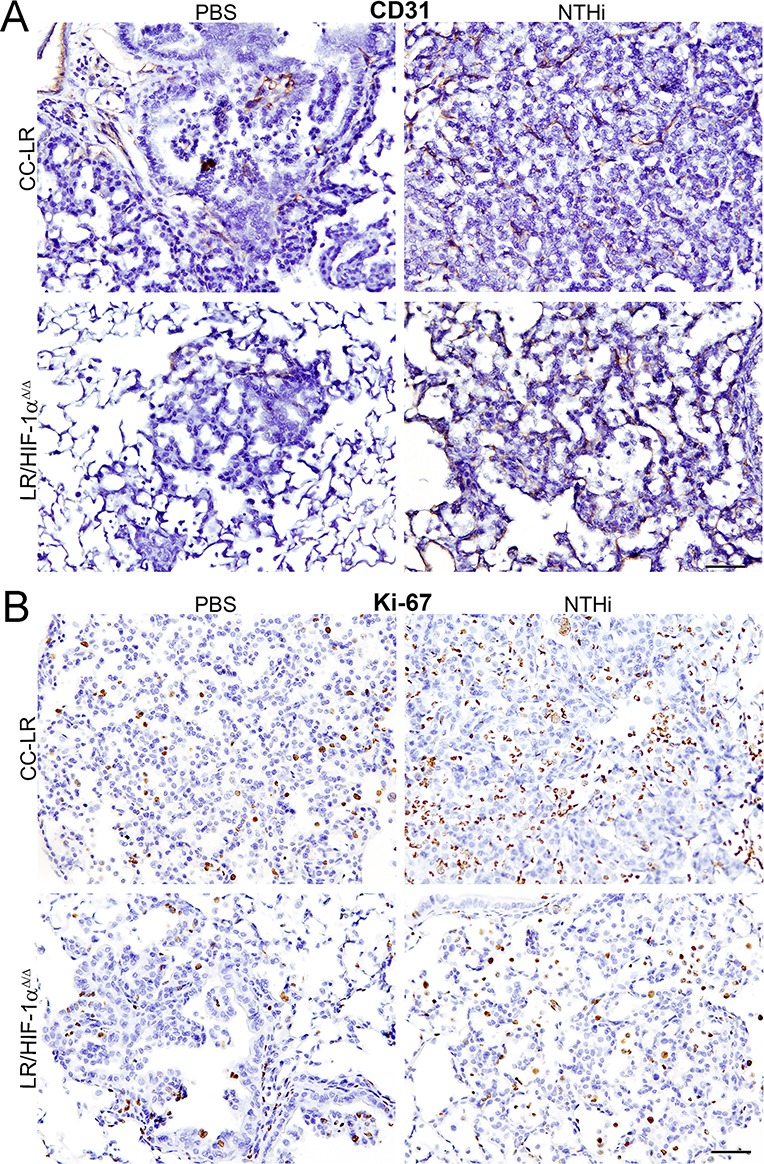
Lack of HIF-1α in the airway epithelium suppresses tumor cell proliferation and angiogenesis Representative photomicrographs of immunohistochemically stained lung tissue from CC-LR and LR/HIF-1α^Δ/Δ^ mice in the absence or presence of NTHi-induced COPD-type airway inflammation at age of 14 weeks for angiogenesis marker, CD31 (**A**), or proliferation marker, Ki-67 (**B**) (20× magnification, scale bar = 50 μm, applicable to all panels).

To study the mechanism of tumor reduction in mice with a lack of epithelial HIF-1α activity, lung tissues were examined for angiogenesis, proliferation, and apoptosis using specific markers. CD31 as well as ERG staining of the lung from LR/HIF-1α^Δ/Δ^ mice showed decreased microvessel density and reduced angiogenesis (Figure 3A, and Supplementary Figure 2A) along with reduced tumor cell proliferation (Figure 3B, and Supplementary Figure 2B) with no changes in tumor cell apoptosis (data not shown) compared to CC-LR mice in the presence or absence of NTHi-induced COPD-like airway inflammation.

### Overexpression of HIF-1α in the airway epithelium causes emphysema and promotes lung cancer

**Figure 4:**
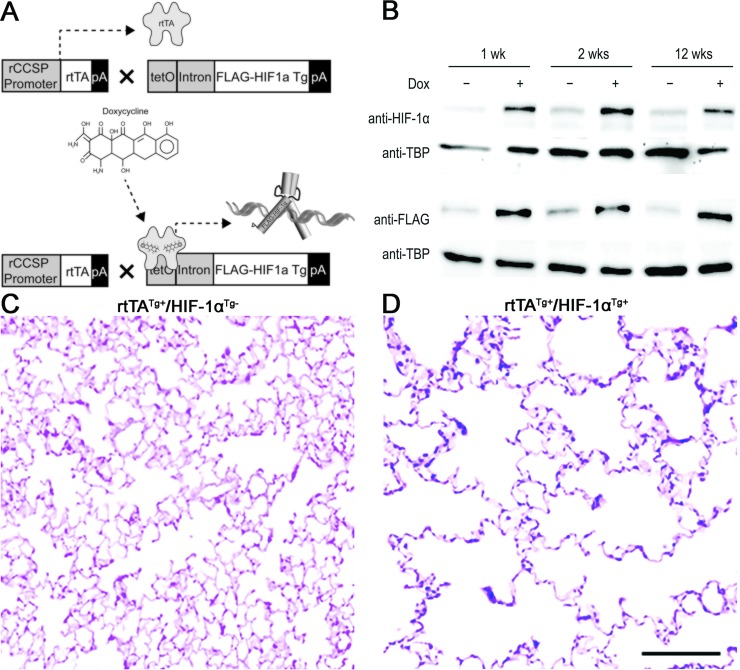
Structure and effect of the HIF-1α transgene expression in the airway epithelium (**A**) Schematic representation of HIF-1α transgene construct and transgenic mouse development. (**B**) Immunoblotting to confirm endogenous and transgenic HIF-1α protein production. (**C**, **D**) Representative photomicrographs of H&E stained lungs from HIF-1α transgene negative and positive mice showing airspace enlargement and alveolar wall destruction after doxycycline treatment in transgene positive but not negative mouse.

**Figure 5:**
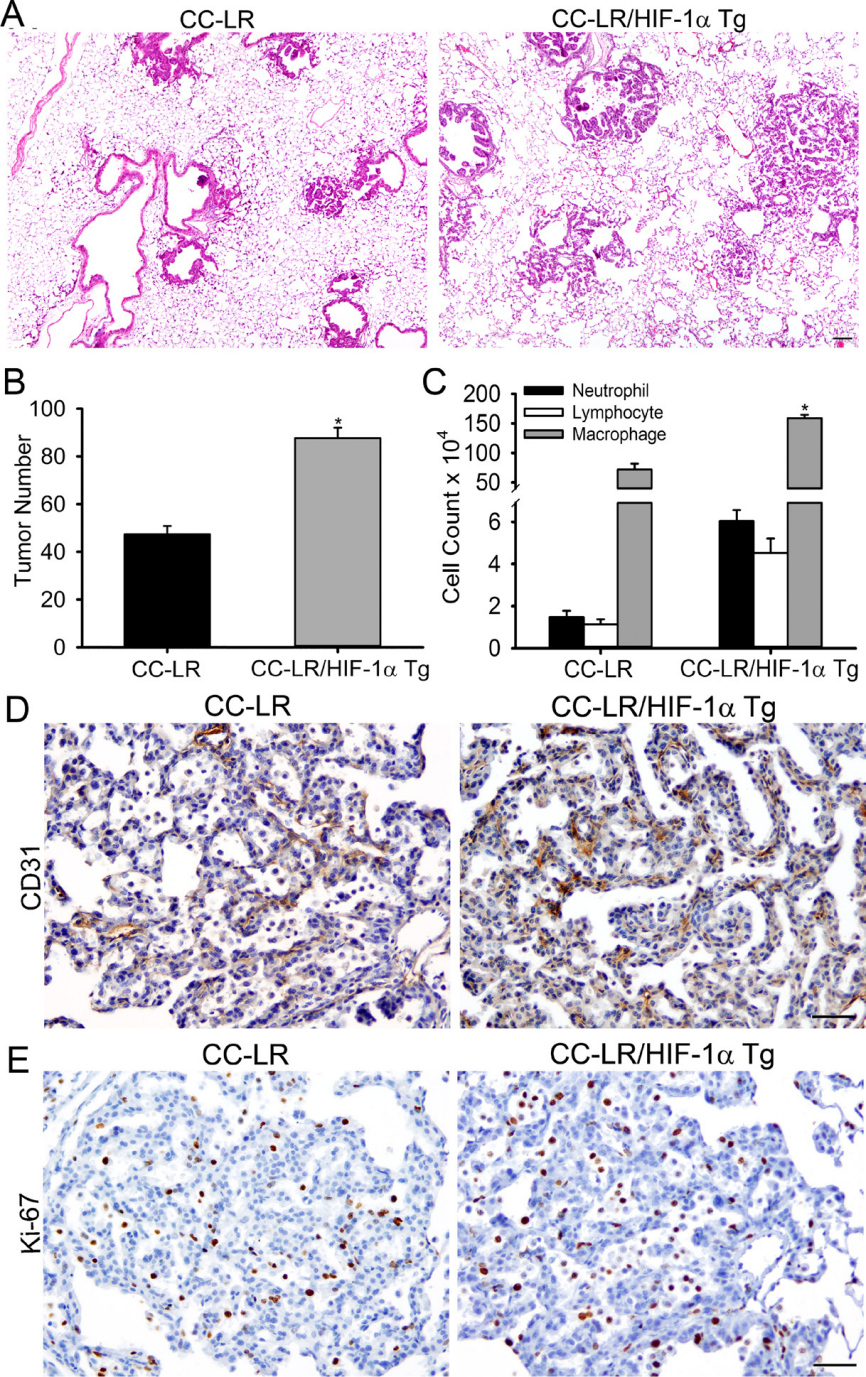
Overexpression of HIF-1α in the airway epithelium promotes K-ras induced lung tumorigenesis (**A**) Histopathological appearance of lung tissue from CC-LR and CC-LR/HIF-1α Tg at age of 14 weeks (4× magnification, scale bar = 10 μm, applicable to all panels). (**B**) Lung surface tumor number in CC-LR and CC-LR/HIF-1α Tg mice at age of 14 weeks (mean ± SE; ^*^, *P* < 0.05 for CC-LR vs. CC-LR/HIF-1α Tg; *n* = 8). (**C**) Lineage-specific leukocyte numbers in BALFs of CC-LR and CC-LR/HIF-1α Tg mice at age of 14 weeks (mean ± SE; ^*^, *P* < 0.05 for CC-LR vs. CC-LR/HIF-1α Tg; *n* = 4). (**D**, **E**) Representative photomicrographs of immunohistochemically stained lung tissue from CC-LR and CC-LR/HIF-1α Tg mice at age of 14 weeks for angiogenesis marker, CD31 (D), or proliferation marker, Ki67 (E) (20× magnification, scale bar = 50 μm, applicable to all panels).

To further confirm the promoting role of HIF-1α in lung cancer, we have developed a mouse with overexpression of HIF-1α in the airway epithelium by crossing a CCSP-rtTA mouse to HIF-1α Tg mouse (CCSP-rtTA/HIF-1α Tg mouse) (Figure 4A, 4B). Interestingly, this mouse (rtTA^Tg+^/HIF-1α^Tg+^), when treated with doxycycline starting at the age of 6 weeks, developed an emphysematous lung phenotype with alveolar wall destruction and an abnormal enlargement of alveolar spaces compared to its transgene-negative control littermate (rtTA^Tg+^/HIF-1α^Tg−^) (Figure 4C, 4D, and Supplementary Figure 3), suggesting a role for HIF-1α in COPD pathogenesis. The CCSP-rtTA/HIF-1α Tg mouse was then crossed to the CC-LR mouse for development of a mouse with mutant K-ras expression and overexpression of HIF-1α in the airway epithelium (CC-LR/HIF-1α Tg) when treated with doxycycline. A cohort of CC-LR/HIF-1α Tg mice was treated with doxycycline chow from the age of 10 weeks for 4 weeks, then their lungs were studied for inflammation and tumors. Overexpression of HIF-1α resulted in a significant increase (~50%, 1.8-fold) in lung surface tumor number in age and sex-matched CC-LR mice treated with the same amount and duration of doxycycline (47 ± 3 in CC-LR 87 ± 4 in CC-LR/HIF-1α Tg) (Figure 5A, 5B), which is less than the promoting effect of NTHi-induced COPD-type airway inflammation in the CC-LR model (~3.2-fold) as we have previously shown [22]. This was associated with a significant increase in levels of IL-6 and KC (Table 1), and subsequent recruitment of macrophages and neutrophils into the lung (Figure 5C).

Macroscopic and microscopic examination of the lungs from CC-LR/HIF-1α Tg mice showed slightly bigger tumors and the development of more papillary-structured advanced and invasive tumor phenotypes (Figure 5A) with metastatic invasion to the chest wall and intrathoracic lymph nodes (data not shown). No distant metastasis was found. Lung tumors from CC-LR/HIF-1α Tg mice also showed increased expression of angiogenic (Figure 5D, and Supplementary Figure 4A) and proliferation (Figure 5E, and Supplementary Figure 4B) markers, but with no changes in apoptosis (data not shown).

## DISCUSSION

A hypoxic tumor environment leads to necrosis of cells located far from vessels, but activation of HIF-1α in surviving tumor cells closer to the vessels [25]. Later, this results in expression of genes in charge of neoangiogenesis (e.g. VEGFs and their receptors). Meanwhile, necrotic cells release intracellular molecules including alarmins and damage-associated molecular patterns, which trigger a proinflammatory response (intrinsic or tumor initiated inflammation) by activation of the NF-κB pathway that could further amplify the angiogenic signals initiated by HIF-1α activity. On the other hand, just as hypoxia can induce inflammation, inflamed lesions in chronic inflammatory conditions (extrinsic inflammation) often become severely hypoxic as a result of increased metabolic demands or reduced metabolic substrates [26–29]. It is also known that HIF-1 expression is upregulated in response to inflammatory stimuli including IL-1β, TNF, and bacterial products through pathways involving NF-kB, highlighting an interdependence of the innate immune and hypoxic responses to infection and tissue damage [28].

Here we have found that K-ras mutant lung tumorigenesis and its promotion by COPD-like airway inflammation is associated with significant tumor angiogenesis and activation of HIF-1α. Hypoxia and HIF-1α activation also potentiate TLR4 expression, enhance the responses of macrophages to LPS, and result in the production of inflammatory cytokines, including IL-6 and TNF [30, 31]. We and others have previously demonstrated that these same signals, in the setting of COPD-type inflammation, promote lung tumorigenesis [22, 23, 32, 33]. We have further shown that deleting HIF-1α in the airway epithelium results in significant tumor reduction and reduced angiogenesis while its overexpression in the epithelium has the opposite effect, resulting in COPD-type inflammation, emphysema and tumor progression. This supports the epithelial HIF-1α pathway activation as an essential process for the transition between COPD and lung cancer. HIF-1α activation was associated with increases in the levels of the inflammatory cytokine IL-6 and chemokine KC, which we have already shown to have essential roles in K-ras mutant lung tumorigenesis and its promotion by COPD-type airway inflammation [23, 34]. Chronic inflammation in COPD could result in local hypoxia in inflamed or remodeled tissues (lungs) via decreased supply (i.e., reduced O2 diffusion through edematous tissue, airway, and thickened mucus or through vascular shunting and decreased O2 delivery to the epithelium), or it could occur via increased demand due to excessive O2 consumption by epithelial cells which ultimately leads to activation of the HIF-1α pathway.

In addition to inflammation and structural remodeling that occur in COPD, HIF-1α expression has also been shown to be increased in basal and goblet cells in the lungs of smokers with COPD compared with healthy smokers, with expression localized almost exclusively to areas of airway remodeling [19, 35], and in positive association with expression of VEGF [36]. In addition, a conserved consensus motif for HIF-1 binding in the promotor of the *Muc5ac* mucin gene has been demonstrated [37], suggesting the possible involvement of hypoxia-inducible signaling in up-regulation of mucus production, a hallmark of lung adenocarcinoma. It has been shown that HIF-1 signaling promotes goblet cell metaplasia and hyperplasia in airway epithelium in an *in vitro* model using human bronchial epithelial cells [35]. Taken together, these data demonstrate a prominent role for HIF-1 mediated epithelial differentiation in COPD and perhaps its transition to lung cancer.

In human non-small cell lung cancer (NSCLC), overexpression of HIF-1 was found in 32.2% of primary resectable tumors and this was associated with reduced patient survival and increased tumor re-occurrence [18]. HIF-1 expression in epithelial cells is seen in all types of NSCLCs, including squamous and adenocarcinoma tumor types, as well as in small cell lung cancer [19, 20]. It has been shown that treatment with a small molecule inhibitor of HIF-1α, PX-478, inhibited progression and spread of orthotopic human small cell lung cancer and lung adenocarcinoma in mice [38]. However, in contrast to our finding, in mice injected with A549 cells, silencing HIF-1α impaired tumor vascularization and increased the necrotic area, but did not reduce tumor cell proliferation and only slightly impacted tumor growth [39]. Note that this study was done using a xenograft model in immune-deficient mice, which does not fully recapitulate the lung tumor microenvironment. On the other hand, it has been found that knockdown of HIF-1α in a human NSCLC cell line results in the restoration of cytotoxic T lymphocyte-mediated tumor cell lysis under hypoxic conditions [40]. The divergent effects of HIF-1α silencing in these studies suggest a complex regulation of tumor growth and invasion that is not controlled simply by the absence or presence of HIF-1, but rather may be dependent on different pathways interacting with the HIF pathway in tumor cells and the tumor microenvironment.

Taken together, we conclude that HIF-1α, an essential transcription factor that is activated in COPD and lung tumors, is essential for the lung tumor-promoting effect of COPD-associated inflammation, and can contribute to lung tumor progression and invasion by providing a microenvironment favoring tumor angiogenesis and tumor cell proliferation. Giving these facts, development of specific agents inhibiting this signaling pathway may represent a novel strategy for immunotherapeutic intervention, and might be beneficial for lung cancer prevention and treatment.

## MATERIALS AND METHODS

### Animals

CC-LR mice (CCSP^Cre^/LSL–Kras^G12D^) were generated as previously described [22]. Briefly, these are mice generated by crossing mice harboring a Lox-Stop-Lox–Kras^G12D^ allele with mice containing Cre recombinase inserted into the CCSP locus; also called secretaglobin-1a1, Scgb1a1; (we will use CCSP for the rest of this paper) [22]. K-ras mutant airway-specific HIF-1α knock out (LR/HIF-1α^Δ/Δ^) mice were generated by crossing CC-LR mice to HIF-1α^F/F^ mice previously generated by Dr. Randalll Johnson [24] and kindly provided by Dr. John Shannon of the Cincinnati Children's Hospital Medical Center. CCSP-rtTA Tg (line 2) mice and tetO-Cre mice were provided by Dr. Jeffrey A. Whitsett of the Cincinnati Children's Hospital Medical Center [41]. These mice were used to generate a transgenic mouse with overexpression of HIF-1α in the airway epithelium as described below. All mice were housed in specific pathogen-free conditions and handled in accordance with the Institutional Animal Care and Use Committee of MD Anderson Cancer Center. Mice were monitored daily for evidence of disease or death.

### NTHi lysate aerosol exposure

A lysate of NTHi strain 12 was prepared as previously described [21], the protein concentration was adjusted to 2.5 mg/ml in phosphate buffered saline (PBS), and the lysate was frozen in 10 ml aliquots at −80°C. To deliver the lysate to mice by aerosol, a thawed aliquot was placed in an AeroMist CA209 nebulizer (CIS-US) driven by 10 l/min of room air supplemented with 5% CO2 for 20 min. CC-LR and CC-LR/HIF-1α^Δ/Δ^ mice were exposed to the lysate starting at 6 weeks of age once a week for 8 weeks.

### Generation of conditional HIF-1α transgenic mice

HIF-1α levels are controlled at both the transcript and protein levels. At the protein level, HIF-1α is tightly regulated by an O_2_ sensitive degradative pathway that involves the activities of prolyl and asparaginyl hydroxylase proteins [17]. These utilize 2-oxoglutarate (2OG, also called α-ketoglutarate) generated during aerobic metabolism as an oxygen donor for hydroxylation of HIF-1α. When O_2_/2OG levels are normal, HIF-1α is down-regulated by functional and degradative means. A human HIF-1α cDNA plasmid construct used previously for overexpression in cell lines [42] was re-engineered to generate doxycycline-inducible transgenic mice by mutating these hydroxylation targets (Figure 4A, 4B). The final construct used for cloning encoded an N-terminal FLAG-epitope tag, followed by a HIF-1α cDNA mutated such that prolines 402 and 564 were substituted with alanine and glycine, respectively, and asparagine 803 was substituted with alanine. These mutations prevent degradation and promote transcriptional activation of HIF-1α (a constitutively active transgene).

A tetO transgenic targeting construct was generated by modifying a transgenic vector we used previously to overexpress TNF in mouse lungs [21, 37]. In this case, the mouse CCSP promoter fragment was excised and a tetracycline operator-cytomegalovirus fusion promoter was inserted in its place. The FLAG-tagged constitutively active HIF-1α cDNA described above was inserted into the tetO-expression cassette using cloned NheI sites, and orientation was confirmed by DNA sequencing. The final targeting construct (Figure 4A) was injected into C57BL/6 pro-nuclei at the MD Anderson Cancer Center Genetically Engineered Mouse Facility.

To induce transgene expression, tetO-HIF-1α Tg mice were crossed with rat CCSP-rtTA Tg (line 2) mice [41]. Resulting progeny were placed on a doxycycline supplemented diet (625 mg doxycycline/kg chow) at defined time points. The doxycycline diet was consumed *ad libito* and changed weekly. To confirm endogenous and transgenic HIF-1α protein production, tissues were disrupted, and nuclear proteins were purified using a NE-PER Nuclear and Cytoplasmic Extraction Reagents kit (Thermo Scientific, Rockford, IL, USA), and 25 μg protein samples were separated on 7.5% SDS-PAGE gels under reducing conditions. Proteins were transferred to PVDF membranes and analyzed by immunoblotting with anti-HIF-1α (1:500, Novus Biologicals, Littleton, CO, USA), anti-FLAG (Sigma, St. Louis, MO, USA), and anti-TATA-binding protein (1:5,000, Novus Biologicals) antibodies. Blots were probed with HRP-conjugated goat-anti-mouse IgG light chain specific secondary antibodies (1:10,000, 2 h, Jackson ImmunoResearch, West Grove, PA, USA), then developed using chemiluminescent Super Signal West Femto HRP substrate detection (Thermo Scientific), and were imaged using a ChemiDoc XRS (Bio-Rad, Hercules, CA, USA). Duplicate gels and transfers were run, so anti-HIF-1α and anti-FLAG antibodies would not overlap (Figure 4B). Anti-TBP loading controls were performed for each blot. Transgenic progeny were subsequently crossed to CC-LR mice for development of CC-LR/HIF-1α Tg mice and placed on a Dox diet as described above. Generation and genotypes of all mouse models used in this study are summarized in Supplementary Table 1.

### Western blot analysis

Total proteins were prepared from each group of mouse lungs as follows. Lung samples were removed and immediately placed in RIPA buffer and a protease inhibitor mixture. The samples were then homogenized and centrifuged at 14000 g for 20 min at 4°C. The supernatants were collected as the total proteins. Nuclear proteins were extracted using NE-PER Nuclear Protein Extraction Kit (Pierce, IL, USA) according to the instructions. Protein concentrations were measured using the Bradford protein assay (Bio-Rad Laboratories). Equal amounts (50 μg for total proteins and 20 μg for nuclear proteins) of the proteins were boiled for 5 min in loading buffer, loaded on each lane, and separated by 10% SDS-PAGE. The gels were then transferred to nitrocellulose membranes. Equal amounts of protein loading for each lane was checked by Ponceau (Sigma Chemical Co., MO, USA) staining. The anti-VEGF, anti-HIF-1α, anti-Lamin-B1 and anti-β-actin (Abcam, Cambridge, United Kingdom) antibodies were diluted to 1:1000. Immunoreactive bands were detected with Pierce ECL Western Blotting Substrate (Pierce, IL, USA).

### Histochemistry and immunohistochemistry

Tissues were taken from mice with the following genotypes: CC-LR, LR/HIF-1α^Δ/Δ^, CCSP-rtTA/HIF-1α Tg, and CC-LR/HIF-1α Tg. Mice were anesthetized and sacrificed by intraperitoneal (IP) injection of avertin (Sigma, St. Louis, MO, USA), then the tracheas of euthanized mice were cannulated with PE50 tubing and sutured into place. The lungs were infused with 10% buffered formalin (Sigma), removed, and placed in 10% buffered formalin for 18 h. Tissues then were transferred to 75% ethanol, embedded in paraffin blocks and sectioned at 5-μm thickness. The sections on glass slides were dried at 60°C for 15 min, and then were deparaffinized and stained with hematoxylin and eosin (H&E) by incubating the tissues in Harris hematoxylin (Sigma) followed by serial eosin (Sigma) and graded ethanol steps. The H&E stained slides were examined by a pathologist blinded to genotype and treatment, and the proliferative lesions of the lungs were evaluated in accordance with the recommendations of the Mouse Models of Human Cancer Consortium [43]. The severity of lung inflammation was also evaluated based on the extent of the lung tissue involvement with inflammatory lesions in H&E stained slides. Alveolar space was measured using the D2 index from 20× magnification lung images from each mouse, ten images each, three mice per group as previously described [44]. Formalin-fixed, paraffin-embedded sections (5 μm) were also labeled with anti-CD31 (1:10, BD Biosciences, CA, USA), anti-ETS-related gene (ERG) (1:1000, Abcam, MA, USA), anti-cleaved caspase 3 (CC3) (1:500, Abcam, MA, USA), and anti-Ki-67 (1:200, Abcam, MA, USA) antibodies after antigen retrieval. Slides were then incubated with biotinylated IgG secondary antibodies specific for each primary antibody followed by incubation with ABC kit (Vector Laboratory, Burlingame, CA, USA) for 30 min, developed with diaminobenzidine (Vector, Burlingame, CA, USA) for 4-10 min, and counterstained with hematoxylin (Sigma-Aldrich). Images were obtained by an OLYMPUS BX 60 inverted microscope at 4×, 10×, 20× or 40× magnification with Image-Pro Plus, version 4.5.1.22. The numbers of labeled positive cells for CD31, ERG, and Ki-67 were quantitated as a fraction of total tumor nuclei per high power field (40×) in 10 fields from three mice of each group. Results were expressed as a percentage of positive cells ± standard error of the mean (SEM).

### Assessment of lung tumor burden and inflammation

On the first day after the final NTHi exposure, animals were euthanized by IP injection of a lethal dose of avertin. In all mice, lung surface tumor numbers were counted, then in some of them, the lungs were prepared for histological analysis as described above. In other mice, BALF was obtained by sequentially instilling and collecting two aliquots of 1 ml PBS through a tracheostomy cannula. Total leukocyte count was determined using a hemacytometer, and cell populations were determined by cytocentrifugation of 300 μl of BALF followed by WrightGiemsa staining. The remaining BALF (~1,400 μl) was centrifuged at 1,250 × *g* for 10 min, and supernatants were collected and stored at −70°C. Cytokine concentrations in BALFs were measured in duplicate by multiplex bead-based assay (EMD-Millipore, Bilerica, MA, USA) using a Luminex 200 analyzer (Luminex Corp, Austin, TX, USA) as detailed previously [45].

### Statistical methods

Summary statistics for cell counts in BALF, and quantitative staining of CD31 in tumors were computed within treatment groups, and analysis of variance with adjustment for multiple comparisons was performed to examine the differences between the mean cell counts of the control group and each of the NTHi treatment groups. For tumor counts, comparisons of groups were made using Student's *t*-test. Differences were considered significant for *P* < 0.05.

## SUPPLEMENTARY MATERIALS FIGURES AND TABLE



## Abbreviations

COPD: chronic obstructive pulmonary disease
HIF-1: hypoxia inducible factor-1
CC- LR: CCSP^Cre^/LSL–Kras^G12D^
LSL: Lox-Stop-Lox
Scgb1a1: secretaglobin-1a1
Dox: Doxycycline
HRP: horse radish peroxidase
IP: intra peritoneal
H&E: hematoxylin and eosin
BALF: bronchoalveolar lavage fluid
NTHi: non-typeable *Hemophilus influenzae*
VEGF: vascular endothelial growth factor
CCSP: club cell secretory protein
NF-κB: nuclear factor-kappa B
LPS: Lipopolysaccharides
IL-6: interleukin 6
TNF: tumor necrosis factor
KC: keratinocyte-derived chemokine
Muc5ac: mucin 5AC
NSCLC: non-small cell lung cancer
