# Supplementary material for: COPD-Type lung inflammation promotes K-ras mutant lung cancer through epithelial HIF-1α mediated tumor angiogenesis and proliferation
Source: Oncotarget. 2018 Aug 31;9(68):32972–83. doi: 10.18632/oncotarget.26030 (PMC6152479; doi:10.18632/oncotarget.26030)
Supplement: Supplementary file 1 [file oncotarget-09-32972-s001.pdf]

COPD-type lung inflammation promotes K-ras mutant lung cancer through epithelial HIF-1α mediated tumor angiogenesis and proliferation

SUPPLEMENTARY MATERIALS

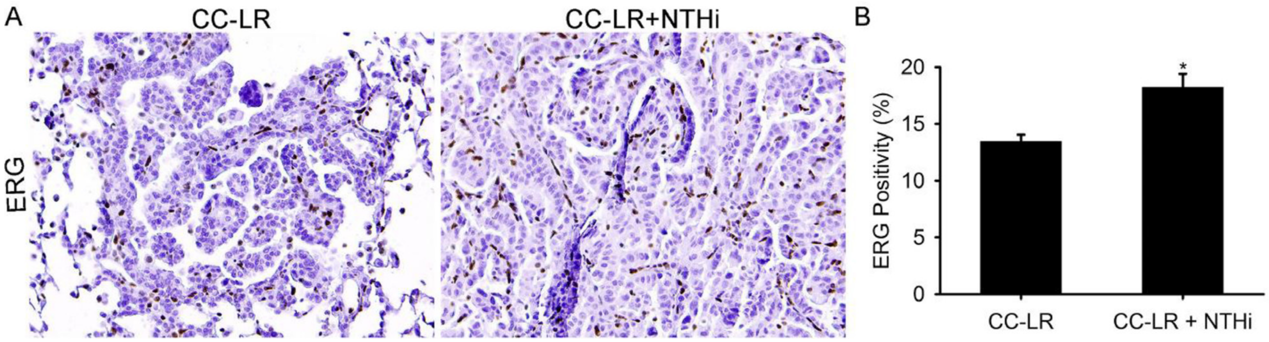

Supplementary Figure 1: (**A**) Representative photomicrographs of ERG immunolabeled lung tumor tissues of 14 weeks old CC-LR mice in the absence or presence of NTHi-induced COPD-type airway inflammation (20× magnification). (**B**) Quantitative analysis of ERG positive staining in lung tissue of CC-LR mice in the absence or presence of NTHi exposure (mean ± SE; * = $P \leq 0.05$ for CC-LR vs CC-LR plus NTHi; $n = 3$).

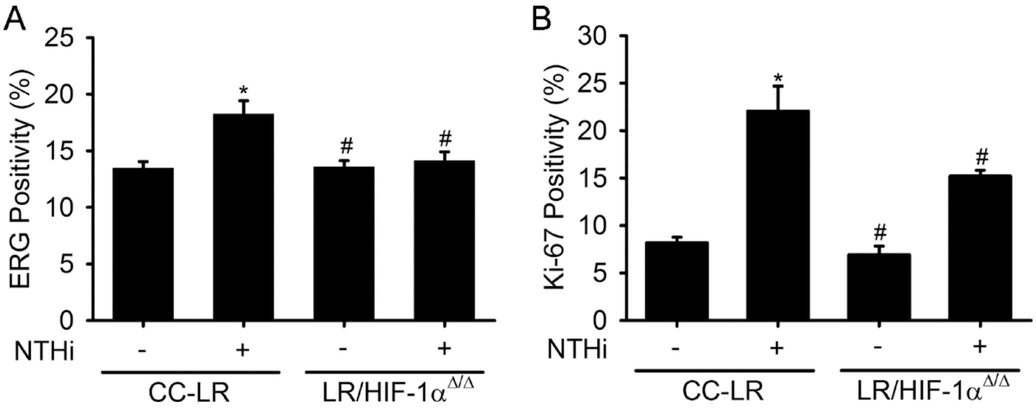

Supplementary Figure 2: (**A**) Quantitative analysis of ERG positive staining in lung tissue of CC-LR and LR/HIF-1α$^{\Delta/\Delta}$ mice in the absence or presence of NTHi exposure (mean ± SE; * = $P \leq 0.05$ for CC-LR vs others, and # = CC-LR plus NTHi vs others; n=3). (**B**) Quantitative analysis of Ki-67 positive staining in lung tissue of CC-LR mice in the absence or presence of NTHi exposure (mean ± SE; * = $P \leq 0.05$ for CC-LR vs others, and # = CC-LR plus NTHi vs others; $n = 3$).

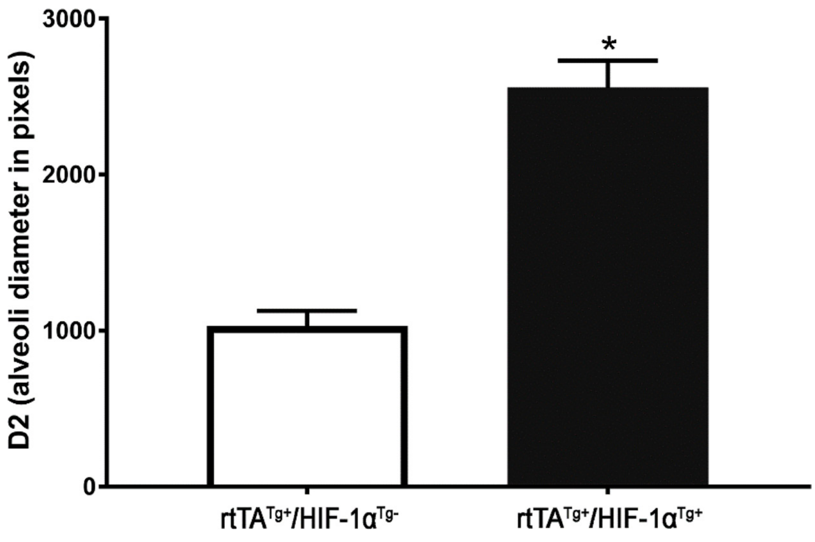

Supplementary Figure 3: Quantitative analysis of alveolar space diameter in lung specific HIF-1α transgenic mouse (mean ± SE; * = $P \leq 0.05$; n = 3).

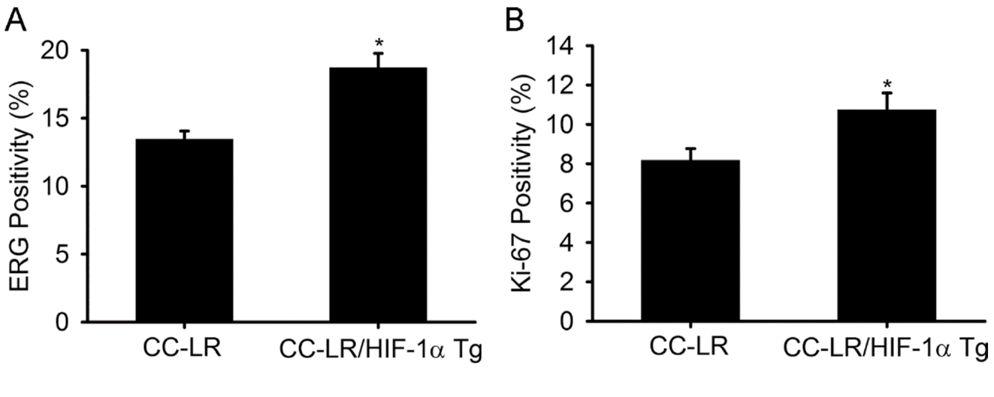

Supplementary Figure 4: (**A**) Quantitative analysis of ERG positive staining in lung tissue of CC-LR and CC-LR/HIF-1α Tg (mean ± SE; * = $P \leq 0.05$; n = 3). (**B**) Quantitative analysis of Ki-67 positive staining in lung tissue of CC-LR and CC-LR/HIF-1α Tg mice (mean ± SE; * = $P \leq 0.05$; n = 3).

Supplementary Table 1: Summary of mouse models

Terminology	Cross	Genotype
CC-LR	$CCSP^{Cre}$/LSL–K-ras^{G12D}	Airway Specific K-ras Mutant
LR/HIF-1$\alpha^{\Delta/\Delta}$	$CCSP^{Cre}$/LSL–K-ras^{G12D}/HIF-1$\alpha^{f/f}$	Airway Specific K-ras Mutant with lack of HIF-1α
$rtTA^{Tg+}$/HIF-1α^{Tg+}	CCSP-rtTA/HIF-1α Tg	Airway Specific HIF-1α Overexpressor
CC-LR/HIF-1α Tg	$CCSP^{Cre}$/LSL–K-ras^{G12D}/CCSP-rtTA/ HIF-1α Tg	Airway Specific K-ras Mutant with HIF-1α Overexpression
